# Ecosystem Vulnerability. New Semantics for International Law

**DOI:** 10.1007/s11196-023-09998-7

**Published:** 2023-04-28

**Authors:** Mariano Longo, Vincenzo Lorubbio

**Affiliations:** Department of Human and Social Sciences, University of Salento, Lecce, Italy

**Keywords:** Vulnerability, Vulnerable persons, Risk, International Law, Human Rights, Rights of Nature

## Abstract

The effects of climate change and increasing environmental pollution have clearly shown the vulnerability of individuals, local communities, and the natural environment, even in the Western context. However, despite such unquestionable data, International Law is still struggling to find adequate, unambiguous, effective solutions to the issue. Even the ‘human right to a healthy environment’, recognised by the UN General Assembly in 2022, is permeated by an anthropocentric idea of the world, which prevents it from fully dealing with ecosystem issues so as to protect any living and non-living being. The paper starts by exploring the historical relevance of the concept of limit and the lack of boundaries in contemporary society, aiming to show that new semantics are needed, in order to overcome contemporary extractivism. An analysis of international legislation and jurisprudence will investigate the role that the concept of ecosystem vulnerability might play in the implementation of both human rights and the rights of nature.

## Introduction

As modern society is characterised by an absence of boundaries in a variety of fields, including science, law, ethics, and technology, limits are set only to be overcome. A feature of modernity, boundlessness may be better understood as a character of contemporary capitalism, which is limitless in terms of both geographical space (globalisation) and functioning (constantly needing to foster endless growth). The lack of limits legitimises an agonic approach to nature, culturally seen as being capable of setting obstacles to be overcome in order for society to pursue new, never-ending tasks [1]. Such an agonic relationship with nature seems to be the most remarkable feature of the Anthropocene.

The relevance of boundaries in other historical and cultural contexts may be shown through an analysis of the Latin concept of *lucus*, which exemplifies the normative relationship between society and the environment. Most archaic societal organisations stressed the cultural relevance of limits and boundaries even when dealing with the environment. Boundaries were defined within which specific prohibitions were effective. In Ancient Rome, the legal protection of the environment was enforced by the concept of limit, interpreted as a geographical, ethical, and religious notion. The best example of that was the sacred grove (*lucus*, specified as *lucus sacer* in the imperial period), a well-delimited space where specific norms and bans were legally effective [2, 3].

A *lucus* could be sacred due to it being either devoted to a god or adjacent to a religious building. In the first case, gathering natural resources was allowed only at well-defined ritual conditions; in the second, only the caretakers of the sacred building could benefit from what the grove had to offer [2, 112]. Therefore, the religious and legal protection of a sacred grove (a praxis shared by most Indo-European cultures) was not determined by human choices, but it was the translation of its sacredness into public law norms. A *lucus* was protected for religious reasons, with a *genius loci* being supposed to preside over it. The protection of a *lucus* may hence be explained by the specific representation of the natural world that characterised pre-modern societies [2].

However, the aforementioned practice does not mean that the Ancient Romans did not exploit the environment. It is well known that they were able to radically transform the overall geography of a place in order to exploit mineral resources, an example of which is provided by the case of the gold mines of Las Médulas in Spain [4]. As Plinius the Old stated, consumption models could lead to the extinction of species, which is what happened with the silphium, an aromatic plant that died out due to its extensive use in Roman cuisine [5]. Yet, the concept of limit was culturally active, at least as a premise: nature possessed an intrinsic sacredness, demonstrated by the alleged presence of tutelary deities. Rather than being determined by political decisions, as it is in the case of modern natural parks, the value of sacred places was intrinsic. As the value of sacred places was intrinsic, their legal protection was perceived as a binding duty, hence independent from human will [6]. This cultural representation of nature set limits to human activities and societies. And insofar as social actors accepted those limits as culturally plausible, legal norms had to be created to define the appropriate human behaviour towards nature.

## Subject, Domination and Risk

It may be argued that the subjectivation of law that characterises modern legal systems could be the premise of a reckless exploitation of nature. It has been maintained that ancient societies, and even Ancient Regime societies, could hardly conceive of subjective rights. Interpreted as privileges, rights were prerogatives of specific social categories, rather than of individuals as such [7, 8]. In the Middle Ages, one could vindicate a right by virtue of their social status (e.g., nobility or clergy), geographical location (citizen or person living in a fiefdom), ethnic origins (Latin or German) and gender (male or female). Legal pluralism was – so to speak – a consequence of the relevance of pre-juridical differences, which still could not converge with the abstraction of the modern legal subject [9].

The modern subject of law is based on a different anthropological conception: endowed with rationality, human beings are considered equal – at least formally – regardless of their social status. Nonetheless, the abstract rationality of the legal subject is fictive, since it overlaps with the anthropological model that was developed by the science of economics in the 19th century and is at the basis of capitalism as a mode of production [10]. The model allowed the political and legal discourse of modernity to conceal actual social differences, in terms of class, ethnicity, and gender, thus producing new modes of domination. The development of modern societies may lead to identify an overall process by which rationality, conceived of as an instrument of human emancipation, converts into an instrument of power and dominion – one only need think of the French Revolution. Indeed, in the historical evolution of Western rationality, the control that the rational subject should have exerted over the world has converted into a form of dominion of social and economic systems over the subject.

The metamorphosis of rationality has been described in a gloomy yet effective way by the thinkers of the Frankfurt School, being thoroughly explored by Horkheimer and Adorno [11]. In *Dialectic of Enlightenment*, the two authors criticise the Enlightenment as a reduction of Western Reason to pure technique, functional to the development of capitalism, interpreted as a form of exploitation of both human beings and nature. Initially an attempt to free human beings from myths, Enlightenment itself transformed into a myth: the myth of human control over the world [11, 6 ff.]. Such a dominion was meant to be double, as it was to be exerted over both apparently rational, emancipated human beings and nature. No longer considered an external and uncontrollable power endowed with a spirituality of its own (as the *lucus* was), nature was seen as tameable and exploitable according to human and social needs. The dominion over human beings and that over nature overlapped, as they were subject to the same processes of economic exploitation [2, 11]. Capitalism evolved as a manipulative system, characterised by limitless exploitation of autonomy, sense, human beings, and natural resources.

However, the introduction of the notion of risk began to challenge the limitlessness of contemporary society, and hence the early modern idea of rationality interpreted as absolute, controlling both the human and natural world. In the late 1980s, Ulrich Beck [12] started considering risk one of the main features of late modernity, currently also described as ‘risk society’. Following Beck’s approach, risk has become a constitutive feature of everyday experience, being connected with modern industrial production and the use of technology, chemical substances, and innovative processes (e.g., genetic engineering), whose consequences are utterly unknown [13].

Risk is both global and democratic, as every human being is exposed to its consequences, which makes geographical and class differences less relevant than they were in the past. As risks are increasing and spreading, the old social differences associated with social status and national belonging can no longer favour privileged individuals and territorial contexts. Environmental disasters, for instance, do not recognise social status differences nor do they respect national boundaries [12]. Risk has to do with the unpredictability of the unintended consequences of rational decisions made within specific social systems [14]. Such a feature of risk clearly shows the crisis of instrumental rationality, with the output of decision-making processes becoming uncontrollable. By demonstrating the weakness of instrumental rationality, risk imposes a reintroduction of the concept of limit in public discourse [14, 225]^1^. As every human being is democratically exposed to the consequences of decision-making processes and technology and as those processes and technologies may affect both human beings and the environment, the concept of risk shows the strong ties between human beings, and those between human society and nature, thus underlining the relevance of vulnerability as a shared feature – ecosystem vulnerability.

## Environmental Law and Ecosystem Vulnerability

A political reaction to risk society might entail an increase in individual rights at both a national and international level, which may be considered the last chance to implement what Juergen Habermas has called “the unfinished project of modernity” [15]. As risk has shown the fallible nature of rationality, a balancing strategy might be an increase in individual rights, within a logic of unlimited growth that resembles the boundless nature of contemporary capitalism. Such an incremental logic may include – at least formally – new categories of human subjects, apparently giving voice to those who have been so far excluded. Yet, reference is always made to human species, which means creating law in the form of new human rights.

Some scholars have pointed out that the protection of the environment should entail the acknowledgment of specific human rights – environmental human rights [17]. The environmental crisis has affected any human being and society, with its effects having an even more negative impact on some specific groups of individuals (such as climate migrants), which has clearly shown the interconnection between individual needs and interests, dignity, and the preservation of nature [17, 89, 18, 19]. Nonetheless, the category of human rights implies a neat separation between the human and the non-human: human rights refer, by necessity, only to human beings, thus failing to take into account the biosphere in which human beings, as a species, live and develop. The excluded third is nature, together with its legal relevance, which has to be mediated by the interests of specific human individuals or groups. The non-human (e.g., the natural environment) is hence perceived as being worthy of legal protection only insofar as it is connected with specific human needs, such as health and well-being.

Yet, ecology as a science has shown that the vulnerability of the human species is intertwined with the wholesomeness of the natural environment. Therefore, a need for new semantics is gradually emerging, which might result in new laws including the natural environment *per se*, rather than just in relation to human well-being. Although conceptual adjustments have sometimes been considered, with reference being made to the need to protect “*the entire vulnerable living order”* [19, 90], the substantial anthropocentrism of contemporary legal systems has usually been retained. The conceptual weakness of environmental human rights may be blamed on their promoting a sort of juridical speciesism, by which rights have always to be guaranteed to rational subjects, hence to human beings, who are at the top of the species pyramid [20, 24].

Being connected with the finitude of human and non-human beings, the condition of vulnerability may be considered both a theoretical starting point to criticise neoliberal policies and a conceptual tool that may be adopted to single out new categories deserving of juridical inclusion. Vulnerability refers, first and foremost, to an ontological condition, as living beings are all vulnerable. However, it may become specific when it is connected with a certain number of contingent circumstances experienced by specific social groups [21, 155–156]. According to Martha Fineman, who introduced the concept within contemporary legal thought, vulnerability is both a universal quality and a context or situational condition [22, 23]. Following Fineman’s work, vulnerability has been thematised in the wide field of social sciences, which has made a relevant semantic turn possible. The Western conception of the paradigmatic legal subject as an abstract, self-sufficient, rational individual, able to control themselves and the environment, has given way to a new model – a concrete, contextualised individual, needing mutuality and support, and connected with the social and natural environment.

The new vulnerable subject is to be defined not only in relation to other human subjects, but also within the biophysical context (the natural environment) in which they partake. A relevant semantic change is here at work: as the condition of vulnerability is shared by humans and the environment, the concept may be adopted as a theoretical tool to de-subjectivize subjective environmental rights, now extendable to the bulk of living organisms [24, 193] or even to the ecosystem as such [25, 29]. The shared awareness of ecosystem vulnerability may result, at least theoretically, in the overcoming of the traditional conception of the legal subject, providing a conceptual tool for a reconceptualization of the juridical relevance of the natural environment.

By being exposed to risks, individuals experience their frailty. Even societies, whose main task is to ensure the safety of individuals and groups, show their weaknesses when having to deal with risk and emergency protection [4, 26]. One of the lessons the recent Covid-19 pandemic has taught the world is that science and technology do not represent a safe shield against the unexpected. The unpredictability of risk contributes to showing the lack of a hierarchical control over the consequences of human political and juridical decision-making processes: individuals are vulnerable, but so are also social systems and societal organisations, at least whenever they have to face uncontrollable or only partially manageable phenomena [26]. The idea developed by solid modernity [27] that rationality enables human beings to control the social world and the natural environment gives way to a new awareness of human limitedness, which would be faced more serenely only if individuals became aware of being part of complex (social and ecosystem) networks.

Therefore, vulnerability may lead to the overcoming of those meaningless dichotomies, including that between anthropocentrism and ecocentrism [28], which are based on a failure to acknowledge the strong interrelationship between human beings, their societies, and nature.

Law acknowledges legal subjectivity to public and private bodies (hence non-human entities), thus attributing quasi-human characteristics to collective structures. In the process of simulation of human characteristics, legal capacity often facilitates economic processes and accelerates mechanisms of exploitation and consumption of natural resources [4, 20]. Yet, the idea of guaranteeing legal subjectivity to nature as such is strongly debated. Considering nature a legal subject *per se* [29] would be a conceptual innovation implying a radical overcoming of the representation of humans (*qua* individuals and *qua* collective entities) as placed at the centre of the world. The concept of ecosystem vulnerability could accelerate the acknowledgment of nature as the holder of specific rights [29, 50]. A new ecological awareness of the interdependence of society and nature would legitimise a theoretical rejection of the idea of nature embraced by modern law, which sees it as an object at the disposal of human beings and societies. Nature as such would acquire legal capacity, while each human being would be entitled to make use of their capacity to protect the interests of nature, in their own interest to survive [30, 250].

In order to cope with contemporary environmental issues, new semantics and new legal concepts are needed. As the global dimension of the current environmental crisis has been widely acknowledged, an analysis of the relevance of environmental questions within International Law will be carried out in the following Sections, in terms of both legislation and jurisprudence. A global crisis calls for global solutions and therefore a new, global concept of both the subject of law and fundamental rights is required.

## The Role of International Law

The international community has started to deal with environmental issues only since the 1960s [31, 1621]. It was then that environmental degradation was first acknowledged, with the environment being no longer seen as an abstraction, but rather as “the living space, the quality of life and the very health of human beings, including generations unborn” [32, 241–242].

The current international and supranational jurisprudence and regulatory instruments aimed at preventing environmental degradation will be analysed in the following sections, in order to understand whether they are also adequate to protect “ecosystem vulnerability” – that condition of vulnerability that is shared by both the human and non-human beings of the ecosystem.

Despite being widely investigated at a theoretical level [33–36], the connection between environmental degradation and the violation of human rights has barely found an appropriate international regulatory framework, being hence inadequately addressed in the jurisprudence. Nonetheless, over the past decades, the clear interrelation between the effects of climate change and the vulnerability of the ecosystem has emphasised the need to consider the human being-natural environment dyad to be inseparable, even in terms of legal protection.

Following the Rio Conference in 1992, various international Conventions were adopted, including the Convention on Biological Diversity (CBD), the United Nations Convention to Combat Desertification (UNCCD), and the United Nations Framework Convention on Climate Change (UNFCCC), which are all based on the principles of prevention and precaution. Paradoxically being the only non-binding one, the most significant of such instruments is the UNFCCC, which was ratified by all member States of the United Nations and aims at the “stabilization of greenhouse gas concentrations in the atmosphere at a level that would prevent dangerous anthropogenic interference with the climate system” (Art. 2). Said Convention is the first to explicitly describe “humankind”, “food production”, “small island countries”, “countries with low-lying coastal”, “arid and semi-arid areas or areas liable to floods, drought and desertification”, “developing countries” (Preamble) as being vulnerable to the adverse effects of climate change. However, no other definition of “vulnerability” is mentioned, with the concept failing to be emphasised.

Despite that, the UNFCCC can be said to have been a turning point in the fight against climate change, as it paved the way to a number of subsequent documents that were adopted during the annual meetings of the Conference of the Parties (COP). The latter include the Kyoto Climate Change Conference (COP 3) in 1997, and the Paris Climate Change Conference (COP 21) in 2015, when the Paris Agreement was adopted. By signing said Agreement, States Parties committed to keeping global warming below 2 °C, and possibly within 1.5 °C, gradually but drastically reducing their emissions to achieve “net-zero” emissions by 2050. The Paris Agreement opened a new phase of international environmental law by making explicit reference to the protection of human rights in the context of the fight against climate change and the environmental crisis as a whole.

The Preamble to the Paris Agreement clearly states that, when taking action to address climate change, States should “respect, promote and consider their respective obligations on human rights, the right to health, the rights of indigenous peoples, local communities, migrants, children, persons with disabilities and people in vulnerable situations and the right to development, as well as gender equality, empowerment of women and intergenerational equity”. This has meant the international community explicitly recognising that climate change poses a threat to the full enjoyment of human rights and that any action to tackle the issue should take the respect of said rights into account [22, 37]. Such a statement is based on the observation of an increasing overlap between human rights and the content of environmental regulations, as well as highlighting the potential benefits of using mechanisms of human rights protection in the application of environmental regulations [38, 447].

However, despite clearly mentioning the need to protect human rights and focusing on the most vulnerable groups, the Agreement lacks practical implications [39, 61], since no substantial obligations are imposed on the parties in terms of environmental human rights [40, 184]. Therefore, although the parties are urged not to undertake actions that might compromise the enjoyment of human rights, they are not encouraged to take measures that may lead to their full implementation or that may prevent others from interfering with said rights. Proof of this is the fact that the Agreement makes use of general terms, such as “promote” and “consider”, which fail to entail any specific action, rather than more imperative verbs like “protect” and “fulfil”, thus prioritising procedural over substantial obligations, behaviour over results [40, 191–200].

Regardless of the assessment of the Paris Agreement, climate change is undeniably “one of the greatest threats to human rights of our generation” [41]. For this reason, in 2018, the Special Rapporteur on human rights and the environment peremptorily stated that “a safe, clean, healthy, and sustainable environment is necessary for the full enjoyment of a vast range of human rights […]” [2, 42], and that, simultaneously, “the exercise of human rights […] is vital to protection of the environment” [2, 42].

In 2021, following long negotiations, the United Nations Human Rights Council finally recognised the human right to a clean, healthy, and sustainable environment [43]. A year later, with 161 votes in favour and eight abstentions (including China, Russia, and Iran), the UN General Assembly adopted a symmetrical Resolution, declaring access to a clean, healthy, and sustainable environment to be a universal human right [44]. Hailing the Resolution, the Secretary-General of the United Nations stated that it was going to “help […] empower people, especially those that are in vulnerable situations” [45], urging States to accelerate “the implementation of their environmental and human rights obligations” [45]. Furthermore, having reiterated that the Intergovernmental Panel on Climate Change estimates that 3.3 billion people are highly vulnerable to the impact of climate change, the UN High Commissioner for Human Rights declared that “climate action can only be fully effective when it integrates the perspectives of people in vulnerable situations” [46]. Ultimately, the guidance of the United Nations seems to be unambiguous: within the context of the environmental and climate crisis, an effective – rather than ephemeral – protection of human rights cannot disregard the actual condition of vulnerability of all subjects.

## Human Rights and Vulnerability in Europe

### Council of Europe

Although the European Convention on Human Rights (ECHR) does not set out a specific right to a healthy environment, as the European Court of Human Rights (ECtHR) has reiterated over the years^2^, increasing reference to the ECHR has been made by individuals and activists, in order to raise awareness of both environmental issues and the legal implications of human rights protection. Environmental contamination and changes caused by harmful industrial emissions, hazardous waste, and noise pollution may either directly or indirectly impact one’s health and well-being, while violating some of the rights enshrined in the ECHR^3^.

The ECtHR has ruled on more than 300 cases concerning environmental issues. Some of such judgements have contributed to strengthening protection from harm caused by environmental degradation at a national level^4^, while others have not produced any significant changes^5^. However, the acknowledgement of pollution as a phenomenon per se still fails to correspond to the acknowledgement of a violation of an applicant’s rights. Even though in some cases the ECtHR has held that pollution inevitably made those exposed to it more vulnerable to various diseases (*Cordella v. Italy*, § 105), in order for the violation of a right to be recognised, it is necessary to demonstrate that an individual’s private or family sphere – rather than *just* the environment – has been detrimentally affected (ibid., § 101).

A new regulatory approach to environmental issues may soon be adopted by the Council of Europe, with measures characterised by a new reflection on the relationship between human rights, the environment, and vulnerability. In 2021, various resolutions were adopted by the Parliamentary Assembly of the Council of Europe (PACE). The first of said resolutions states that the standards of protection of the right to a safe, clean, and healthy environment are not the same across Europe, in terms of both the subjects that hold such a right and the countries in which it is guaranteed. Poorer countries and the most disadvantaged groups are more detrimentally affected by climate change [47, § 8). The groups mentioned in the resolution include women, children, the elderly, sick persons, minorities, and the poor. They all are caught up in a “vicious circle” of multiple discrimination, as their general condition of vulnerability is worsened by their unequal access to environmental rights. The resolution clarifies that any new legally binding instrument on the right to a safe, clean, and healthy environment should “address all of the sources of inequality […], with the aim of minimising inequalities” [47, § 16]. In order to achieve such an objective, the well-established “four Ps” mechanism should be used, providing for “prevention”, “protection”, “prosecution” and “policies”, which should be complemented by a fifth action – “parliamentary commitment” [47].

A second resolution focuses on human rights protection for those who are forced to migrate due to climate change-induced disasters or hardship, with particular attention to vulnerable groups. It emphasises the need for the introduction of a new instrument that may ensure the human right to a healthy environment [48, § 5]. Such a new instrument may be an additional Protocol to the ECHR, as a third resolution suggests, so as to “anchor” the right to a healthy environment to the non-disputable basis provided by the Convention [49, § 7]. Said third resolution underlines how the absence of specific regulations has resulted in the ECtHR adopting “an anthropocentric and utilitarian approach” to the environment that has prevented natural elements from being afforded any protection per se [49, § 7]. Drafting an additional protocol to the European Social Charter on the right to a healthy environment might also be useful [49, § 10].

The Council of Europe’s system of human rights protection may soon lead to the identification of one or more additional instruments – a new specific Convention, a protocol to the ECHR, a protocol to the European Social Charter – that might make the right to a healthy environment effective, emphasising the aspects associated with the dynamics of environmental degradation, and especially vulnerability and discrimination, “based on the UN guidance on this matter” [49, 14.1].

### European Union

Despite the presence of a regulatory framework that might contribute to the implementation of environmental human rights, none of the instruments of the European Union makes explicit reference to such an option, nor is the relationship between human rights protection and the climate change-induced circumstances of vulnerability thematised. Due to such a persistent difficulty, the European Parliament has prioritised the improvement of the implementation of European environmental law in all the member States. In order for this to happen, Parliament is actively participating in the discussion of the proposals put forward by the European Commission within the European Green Deal^6^.

The new “European Climate Law” of 2021 [50] clarifies that, besides protecting and enhancing the Union’s natural capital, the European Green Deal aims to protect the citizens’ health and well-being from environment-related risks and impacts, an objective that implies a fair and inclusive green transition [50, § 2].

In 2021, Parliament also adopted a Resolution on the effects of climate change on human rights and the role of environmental defenders. The European Union and its member States have been urged to act “through the adoption, strengthening and implementation of legislation aligned with a comprehensive human rights-based approach to climate action” [51, § 1]. In its most significant passage, said Resolution highlights the need for “a reconceptualisation of the relationship between people and nature that will reduce risks and prevent future harm from environmental degradation” [51, § 10]. Furthermore, the Resolution makes multiple references to vulnerability with regard to “people”, “groups”, and “populations” [51, § B, G, E, O, P, 19, 25, 48, 53, 64), but also “countries”, regions” and “areas” [51, § O, Q]. The vulnerable groups of people mentioned include “women, children, persons with disabilities, the elderly, the poor, indigenous people or people belonging to minorities” [51, § 64].

Yet, despite the European Parliament’s guidance, European Law still lacks a clear framework to which environmental human rights may be anchored. Bridging such a gap would mean giving the appropriate value to the growing relationship between environmental vulnerability and the – either direct or indirect – violation of the subjective legal positions of all the parties, and especially those groups that are generally overexposed to risk. Within this context, a greater role might be played by the future judgements issued by the Court of Justice (ECJ), as to date no reference has been made, not even at an interpretive level, to the correlation between human and (biotic and abiotic) environmental vulnerability, which have nonetheless been described by the judges of the ECJ as significant, though unrelated, aspects [52, 163–165].

## Rights of Nature and Vulnerability in the Inter-American Human Rights System

As it has been pointed out, the anthropocentric approach adopted by Western legal systems is based on a concept of nature that sees the latter as being at the human being’s disposal. Such a *Weltanschauung* has already resulted in the transgression of some of the nine planetary boundaries and may lead to further biophysical thresholds of the Earth system being crossed^7^. The idea of an alleged absence of boundaries (see Sect. 1) has collided with the evidence of their transgression.

Since 2009, the United Nations have fostered “Harmony with Nature”, a programme aimed at the achievement of a fair balance between the economic, social and environmental needs of present and future generations. As a result, 11 reports were issued and 13 resolutions were adopted over a decade, from 2009 to 2022. A report published in 2020 highlighted that, due to the acceleration of climate change and the collapse of ecosystems, the human right to a healthy environment cannot be achieved without securing nature’s own rights first [53].

The UN programme aims to promote a new concept of the human-nature relationship in which the parameters for future political (and legislative) action are no longer exclusively based on human priorities [54]. Although the European Union is still struggling with such an approach, some Latin American systems have already successfully adopted it, with the right to a healthy environment^8^ and the rights of nature^9^ being recognised at a constitutional level [55, 141–172, 53, 517–519].

Within the Inter-American regional context, an important Advisory Opinion of 2017 on “environment and human rights” (OC-23/17, § 57)^10^ provided some clarifications on the content of environmental rights by stating that the right to a healthy environment is protected under Article 26 of the American Convention on Human Rights (Economic, Social, and Cultural Rights). Advisory Opinion OC-23/17 describes the right to a healthy environment as an “autonomous right”, aimed at protecting all the components of the environment, including forests, rivers and seas, as “legal interests in themselves”, even in the absence of the certainty or evidence of a risk to individuals (OC-23/17, § 62). This legal instrument points out that the absence of actual risks to the human being is no impediment to the recognition of the existence of risk to the whole ecosystem. As evidence shows, it is no longer possible to distinguish between “human” and “natural” risk (see Sect. 3).

Having recognised the “undeniable relationship” between the negative effects of climate change and the enjoyment of human rights (ibid., § 47), the Inter-American Court of Human Rights pointed out that the climate crisis is the most dangerous environmental issue for vulnerable groups such as indigenous populations, people living in extreme poverty, children, people with disabilities, and minorities. The Court also mentioned coastal and small island communities, and communities whose survival depends on environmental resources that are at risk of degradation, including the marine environment, forested areas, and river basins (ibid., § 67).

The Inter-American system has undeniably emphasised the connection between the right to a healthy environment and the vulnerability experienced by some groups of people and specific natural contexts. The recognition of such a strong correlation also led to the adoption of the Escazú Agreement in 2018 [57]. Entered into force in April 2021 and modelled on a Convention of the Council of Europe on the same topic (Aarhus Convention), this is the first legally binding regional agreement on human rights and the natural environment. It aims to guarantee the full implementation of the rights of access to environmental information, public participation in the environmental decision-making process, and access to justice in environmental matters, thus contributing to the protection of the right of every person of present and future generations to a healthy environment and sustainable development [57, art. 1].

The Agreement describes vulnerability as an “open category”, since persons or groups in vulnerable situations are “those persons or groups that face particular difficulties in fully exercising the access rights recognized in the present Agreement, because of circumstances or conditions identified within each Party’s national context and in accordance with its international obligations” [57, Art. 2(e)]. The Agreement is theoretically based on an idea of sustainable development that can strike a fair balance between the economic, social, and environmental dimensions [57, 8, 9]. It refutes the “false dichotomy” between environmental protection and economic development, since actual growth “cannot take place at the expense of the environment and the environment cannot be managed if our economies and peoples are ignored” [8, 57]. The guiding principles listed in Article 3 also need to be mentioned, as they are generally recognised principles in international treaty law and are essential for the hermeneutic interpretation of the Agreement provisions. One of such principles is the *pro persona* principle, which is a particularly significant interpretive principle since, in case of conflict, it entails the obligation to opt for the most favourable approach to the protection of human rights^11^.

Although the reasons for such a choice are connected with the objective of strengthening the protection of vulnerable persons, and especially indigenous populations, from the exploitation of natural resources, treaties of such kind still adopt a basic anthropocentric perspective. Despite taking into account aspects associated with both the natural sphere and human issues, even the Escazú Agreement does not seem to mark the adoption of an ecosystem approach. While encouraging a sustainable use of resources, the protection of biodiversity, and the fight against climate change, focusing on vulnerable subjects from a bottom-up perspective that may foster social participation and the respect of the non-discrimination principle, the Agreement fails to recognise the urgent need to protect the natural environment regardless of the risk of immediate and clear harm to the human being.

Therefore, it may be interesting to assess the role that the Inter-American Court will play in the implementation of the Escazù Agreement. Although this is agreement is not a constitutive part of the Inter-American framework of human rights^12^, the Inter-American Court may have a key role in clarifying the meaning of its provisions and the connection between the latter and the rights enshrined in the Inter-American Convention of Human Rights, also in light of the ecosystem approach clearly mentioned in Advisory Opinion OC-23/17.

## Concluding Remarks

The effects of ongoing climate change and increasing pollution have emphasised the vulnerability of individuals, communities, and the natural environment, even in the Western context. Despite such unquestionable data, International Law is still struggling to find adequate, unambiguous, effective solutions to the issue.

The human right to a healthy environment has been internationally recognised and might gradually be introduced in most Constitutions worldwide. However, its anthropocentric character prevents it from fully dealing with ecosystem issues so as to protect any living and non-living being. As some scholars have highlighted: “[e]nvironmental human rights retain the entitled, hierarchically superior human as their main referent and beneficiary while failing to address injustices thereby occasioned” [58, 193].

Although some criticism has been expressed about environmental human rights (see Sect. 3), it is undeniable that using well-established argumentative strategies and the powerful international system of human rights protection, in terms of both legislation and jurisprudence, might ensure an effective protection of the natural environment that may not be achieved with alternative instruments.

Such an awareness calls for the identification of legally-binding solutions requiring that both law- and decision-makers respect the natural environment, even within legal frameworks where conceptualising and protecting actual “rights of nature” still seems to be unfeasible.

The social construction of new semantics is still in its initial phase, with the tight human-nature-society interconnection being not juridically (and judicially) operative yet [59]. Such developing semantics may become effective only if the notion of vulnerability as an objective condition shared by human beings, other living beings, and the natural environment is thematised. Emphasising such a concept seems to be essential to overcome the anthropocentric idea of the centrality of individuals and human societies. This centrality implies a kind of cognitive security that strengthens when interpreted in terms of a distinction between the included and the excluded. Conversely, the awareness of sharing their vulnerability with the natural environment places human beings in the actual flow of the stream of life, in the very middle of natural and social processes. This would make them become mindful of the frailty of the ecosystem in which they partake, whose integrity is put at risk due to their pretension to be central [59, 857].

In the law system, here exemplified in terms of international and supranational law, new concepts are emerging that include the idea of sustainability for future generations, environment, and risk. However, both norms and the judicial process are still based on old logic that fosters, at best, the acknowledgment of environmental human rights, with no conceptual attempt at overcoming the neat distinction between human beings and their natural environment, society and nature [59, 865].

Nevertheless, the above-mentioned Ecuadorian (2008) and Bolivian (2009) Constitutions have shown a new legal approach to the environment, considered from an ecological standpoint rather than from the individualistic perspective of human rights. According to this innovative ecological approach, human well-being is to be connected with all the subjects – society, communities, the physical and biological components of the natural environment – that contribute to determining the complexity of environmental interconnections. By connecting the traditional idea of the Earth and nature as quasi-sacred subjects with a critical attitude towards neoliberal extractivism, such an approach may lead to envisage an increasing number of non-human legal subjects.

Procedural rules are emerging to actualise this new theoretical paradigm. An example of that is provided by the *in dubio pro natura* procedural criterion, aimed at encouraging juridical decisions that may foster the protection and respect of the natural environment [29, 78 ff]. The *in dubio pro natura* criterion shows the need to acknowledge the ecosystem character of the environmental crisis at a domestic, supranational, and international level.

One may wonder whether *pro persona*-centred legislation and jurisprudence can embrace an ecosystem approach so to achieve effective *pro natura* protection. Such a question may have a positive answer. What needs to be reconsidered is not the instrument of human rights per se, but rather the concept of “human”. The abstract idea of a “paradigmatic” human subject that is isolated from any context, theoretically invulnerable, and the “owner” of the natural environment, should be replaced by the concrete reality of the *homme situé* [31, 60]. Being part of both society and a broader ecosystem, the *homme situé* is a rational being who shares a vulnerable condition with the other non-human (biotic and abiotic) components of the ecosystem, becoming their custodian in order to survive. Only such a radical conceptual change may make the protection of the natural environment effective, through either the introduction of new international regulatory instruments or an ecosystem-oriented interpretation of well-established ones.

Environmental emergencies, and especially the climate crisis, have dramatically shown (see Sect. 3) that human rights cannot achieve their original objective of promoting “social progress and better standards of life in larger freedom” (Preamble to the Universal Declaration of Human Rights) without embracing “ecosystem obligations”. Only such an approach may result in a reintroduction of the concept of boundary also in the field of law (see Sect. 1). This would prevent the transgression of further planetary boundaries, thus contributing to the pursuit of what may be described simultaneously as human, social, and environmental justice – ecosystem-oriented, sustainable justice.

## Footnotes

1 The concept of limit was reintroduced in the public and academic debate in the 1970s [16].

2 See ECtHR, *Kyrtatos v. Greece*, Application no. 41,666/98, judgement of 22 May 2003, para. 52; *Hamer v. Belgium*, Application no. 21,861/03, judgement of 27 November 2007, para. 79; *Turgut and Others v. Turkey*, Application no. 1411/03, judgement of 8 July 2008, para. 90.

3 Said rights include the right to private and family life (Art. 8), the right to a fair trial (Art. 6), the right to property (Art. 1 of Protocol No. 1 to the ECHR), the right to life (Art.1), and the prohibition of inhuman or degrading treatment (Art. 3).

4 For instance, following *Karin Andersson and Others v. Sweden*, Application no. 29,878/09, 25 September 2014, the Swedish government changed its plans for the construction of a railway adjoining residential properties. Furthermore, following the *Jugheli* case in 2017, Georgia took measures to control and regulate the activities of a power plant.

5 See *Cordella v. Italy*, Application no. 54,414/13 and 54,264/15, 24 January 2019, concerning the ex-ILVA steel plant in Taranto. In this case, the ECtHR held that Italy had not taken the necessary measures to ensure the protection of local residents from pollution caused by the ex-ILVA steel plant, besides failing to provide effective remedies to secure the depollution of the area.

6 The expression describes a series of strategic initiatives that the European Commission has been implementing since December 2019 in order to foster a “green transition”, with the objective of achieving climate neutrality (net-zero emissions) by 2050. Cf. European Commission, COM/2019/640 final and COM/2020/562 final.

7 The concept of planetary boundaries was theorised in 2009 by Swedish researcher Johan Rockström, who identified nine thresholds that, once crossed, may lead to the extinction of life on Earth (cf. https://www.stockholmresilience.org/research/planetary-boundaries/the-nine-planetary-boundaries.html).

8 Constitution of Bolivia, *Sección I*, “Derecho al Medio ambiente” (Arts. 33–35).

9 Constitution of Ecuador, *Capítulo séptimo*, “Derechos de la naturaleza” (Arts. 71–74).

10 The Inter-American Court confirmed such an approach in *Indigenous Communities of the Lhaka Honhat (Our Land) Association v. Argentina* of 6 February 2020, when it held – for the first time in its history – that the violation of the right to a healthy environment is a direct violation of Article 26 of the American Convention on Human Rights. The Court reiterated that several rights may be affected as a result of environmental problems, which may be felt with greater intensity by certain groups in vulnerable situations. For this reason, States are legally obliged to confront these vulnerabilities based on the principle of equality and non-discrimination (§ 208–209).

11 This clearly shows how an interpretive approach developed within the jurisprudence of the Inter-American Court and based on Article 29 of the American Convention on Human Rights has been adopted in the context of an international agreement.

12 Should a dispute arise about the interpretation of the Agreement and if the dispute cannot be settled through negotiations, the parties can only submit the dispute to the International Court of Justice or go to arbitration in accordance with the procedures established by the Conference of the Parties under Article 19.
